# Development and necessary norms of reasoning

**DOI:** 10.3389/fpsyg.2014.00488

**Published:** 2014-05-23

**Authors:** Henry Markovits

**Affiliations:** Département de Psychologie, Université du Québec à MontréalMontréal, QC, Canada

**Keywords:** development, reasoning, logic, norms, communication, epistemology

## Abstract

The question of whether reasoning can, or should, be described by a single normative model is an important one. In the following, I combine epistemological considerations taken from Piaget’s notion of genetic epistemology, a hypothesis about the role of reasoning in communication and developmental data to argue that some basic logical principles are in fact highly normative. I argue here that explicit, analytic human reasoning, in contrast to intuitive reasoning, uniformly relies on a form of validity that allows distinguishing between valid and invalid arguments based on the existence of counterexamples to conclusions.

The question of the potential usefulness of normative models in understanding human reasoning is a complex one, something that underlies some of the more important debates in the psychology of reasoning. Some of the earliest debates about the nature of human reasoning were explicitly framed around the question of whether human reasoning is essentially “logical” (e.g., Henle, 1962). In these debates, the logical position essentially claimed that humans possessed an inferential apparatus that would (mostly) invariably lead to inferences that corresponded to those found in elementary logic textbooks, reprising Boole’s view that Boolean logic simply described human reasoning. A more nuanced approach to this question was given by Braine’s (1978) theory that claimed that humans possessed certain limited syntactic reasoning procedures that invariable led to “logically correct” inferences (see also Rips, 1983). These inference rules were the product of biological evolution. Finally, Piaget’s theory (Inhelder and Piaget, 1958) made a different claim, suggesting that while children went through stages where their reasoning was constrained by physical and concrete parameters, their development led more or less invariably to the stage of formal reasoning, where logical reasoning is the norm. In fact, Piaget explicitly proposed propositional logic (albeit a modified version of this) as a competence model for formal thought.

Unfortunately for these approaches, empirical research has clearly shown that human inferential performance is highly variable (Markovits, 1985; Overton et al., 1987; Cummins et al., 1991). Many studies have shown that when even educated adults are given what appear to be formally identical arguments, they give difference conclusions. Judgments of deductive validity differ as a function of premise content (e.g., Markovits and Vachon, 1990; Thompson, 1994; Cummins, 1995), and in response to factors such as conclusion believability (Evans et al., 1983). There is little surface evidence that the use of classical propositional logic as a consistent basis for inferential reasoning is very wide-spread, even among highly educated populations. One reaction to these studies has been an attempt to reject the idea that human reasoning is logical at all, by suggesting that much of the inferential apparatus is dominated by biologically based forms of inference. For example, the heuristics described by Tversky and Kahneman (2004) and Gigerenzer and Selten (2001), although differing in many respects provide simple, context-specific forms of rapid inferential reasoning. These heuristics are context dependent, and their use can account for at least some of the variability in human reasoning. However, they do not correspond to a clear model of logic of any kind, although one might suppose (as Gigerenzer explicitly argues) that they are biologically efficient. Similarly, the probabilistic model proposed by Oaksford and Chater (2003, 2007) and Evans et al. (2007) suggests that inferential procedures model the (Bayesian) statistical properties of people’s knowledge of their environment. Such models propose that people process relations in a way that explicitly reflects their personal beliefs, which in turn is at least partly determined by real-world knowledge stored in long-term memory (Oaksford and Chater, 2012). Inferences are thus basically probabilistic, and essentially variable, and translate the real nature of people’s underlying knowledge. The question of whether reasoning of this kind can be cast in terms of a normative model is open, partly because there is not a strong consensus about the way that probabilistic models function (Elqayam and Evans, 2013; Oaksford and Chater, 2013).

Nonetheless, it is worth making one specific point in this context. Probabilistic models propose that people’s inferences are determined by their individual estimations of conclusion likelihood. Since there is no mechanism by which such estimations can be judged as being more or less accurate, a normative model that depends on some external criteria might seem to be impossible to verify. It might, however, be possible to model standard deductive inferences within a Bayesian framework. Deductive reasoning can be seen as an attempt to construct a representation of premises for which there is a shared attempt to maintain some consistent level of internal probability, e.g., a shared belief that the probability of *q*∣*p* for a given major premise is close to 1 (Oaksford and Chater, 2012). In this case, it might be possible to use a normative model in order to evaluate the way that people reason in this constrained system. However, since such an exercise is clearly artificial, and does not generally reflect the nature of real world information, norms of this kind will be correspondingly artificial.

The key point is that probabilistic models of inference essentially depend on what must be idiosyncratic representations of real world probabilities, since they depend on information stored in long-term memory. This is quite critical, since it makes Bayesian norms almost by definition undetectable. Bayesian models are used to understand how people can detect environmental regularities, something that is clearly biologically useful, since it allows some level of anticipation of the specific properties of a person’s immediate environment (Tenenbaum et al., 2006). However, environments can be variable and individual experience will reflect this variability. Thus, probabilistic models produce by their very nature variable outputs that cannot be compared since this variability reflects variability in inputs. Inferential reasoning can be applied across the whole range of experience and probabilistic approaches to inference must then reflect the wide variety of individual experience. Thus, it could be argued that these approaches suggest that human reasoning cannot, even in principle, be described by a normative model (Elqayam and Evans, 2011).

In the following, I will nonetheless attempt to argue that despite variability and the undoubted influence of many forms of heuristics, human reasoning in its conscious component does indeed depend on a simple normative form of basic logic (which does not necessarily correspond to a specific logical model), for both epistemological and developmental reasons.

## WHAT IS A NORMATIVE MODEL?

Before attempting a more specific analysis, it is important to make some initial distinctions. Normative models can be considered in very different perspectives (Elqayam and Evans, 2011). In the following, I will consider that a normative model is a prescriptive description of the *optimal* way that a system should function in order to accomplish its basic goals. It is important to distinguish between such models and descriptive models, which are attempts to describe the actual workings of a given system in a real-life situation. Simple variability is not an indication that a normative model is inapplicable to a given system. However, variability requires showing that the system’s functioning tends towards a normative model when conditions are optimized.

There is one further critical part of any analysis of normative models. Normative models are mathematical or logical abstractions that aim to capture the essential functioning of what are necessarily messy and complex systems. Such models are, by definition, the product of human reasoning, since they are the result of people trying to understand the basic parameters of a specific system. The role of normative models in the understanding of human reasoning becomes double-edged, since such models must not only describe the way that people can optimally reason, but importantly these models must also be able to account for the ability of people to construct these models in the first place.

With this in mind, it is useful to note that many normative models have epistemological underpinnings that are essentially based on standard bivalent deductive logic. Given the increasing importance of Bayesian models, as discussed previously, it is particularly useful to note that Bayesian statistics are derived using such logic. In fact, careful analysis of the *arguments* for probabilistic models clearly shows that these are based not on Bayesian inferences, but on classical logical arguments. If human inferences were uniformly Bayesian, then one would expect arguments to be phrased specifically in terms of degree of belief. However, conclusions that explicitly leave open the possibility that alternative theories have a clear probability of being correct are rarely encountered. In other words, it is important to distinguish between the characteristics of the output of a given model, and the epistemological underpinnings of these models. In most fields, the second part of this equation is basically irrelevant. When discussing characteristics of normative models of human reasoning, this becomes fundamental. In fact, one key component of the argument that will be presented is that a minimal normative model for human reasoning is necessary in order to account for the ability to produce normative models in the first place.

## DUAL-PROCESS THEORIES

There is one final distinction that is important in this context. One response to the clear evidence that people’s reasoning does not consistently conform to logical norms is the idea that there exist two separable inferential systems. One of these is meant to be a major source of variability in reasoning, while the other has at least the potential to reason more logically. Dual process theories (Sloman, 1996; Stanovich and West, 1998; Evans, 2007) postulate that people have two major inferential systems that interact with each other. Such theories have multiple forms and use different criteria to attempt to distinguish between these two systems. There is, unfortunately, no real consensus as to the characteristics or the definition of these two systems (see Evans and Stanovich, 2013, for a recent discussion). However, roughly speaking, these postulate a basically heuristic form of inference, which we is often referred to as System 1, which is presumed to be an evolutionarily primitive system that makes rapid inferences that are automated, contextual, use surface properties of problems, and rely extensively on stored knowledge. Such inferences are low-cost and do not involve working memory capacity. The second system, which postulates a more analytic form of inference, referred to as System 2, by contrast, is conscious, slow, and relatively costly in its use of working memory.

Although there are variable descriptions of the dual process framework, Evans and Stanovich (2013) suggest that one minimalist approach to this distinction is to suggest that heuristic inferences correspond to rapid, autonomous inferential processes, while analytic inferences are characterized by working memory-based processes that support hypothetical thinking. The latter are particularly characterized by cognitive decoupling, allowing inferences that are not necessarily tied to existing knowledge structures (Stanovich and Toplak, 2012). For our purposes, a key characteristic of System 1 inferences is they are intrinsically variable, since they necessarily reflect the idiosyncratic nature of people’s internal representations. Given this intrinsic variability, there is no reason to think that a single normative model would ever be able to capture its properties, at least not within a relatively straightforward model. However, System 2 allows at least the possibility of hypothetical thinking involving some degree of conscious processing of information. If this basic distinction is reasonably accurate, then the ability to even consider the possibility that normative models could exist must be the result of System 2 processing.

## DEVELOPMENTAL EPISTEMOLOGY

With these distinctions in mind, there are two forms of argument for a normative model for System 2 reasoning. The first is an epistemological argument that partly owes its form to Piaget’s work on cognitive development. Piaget in fact referred to his field of study as genetic epistemology. The basic argument, which was derived from Kant (see Henle, 1962) can be stated as follows. In order to adequately process the infinite variety of information that is potentially accessible, the human mind requires some basic categories. Kant assumed that the basic categories were a priori, that is, that they are a basic component of the human cognitive system and are essentially biological. Such an essentialist view of the basis of human cognition is actually quite common in many developmental theories. For example, studies examining people’s understanding of categories and concepts (Gelman and Markman, 1986), and object permanence (Baillargeon et al., 1985) have indeed claimed that people have biological underpinnings that allow them to consistently extract specific categories or object qualities from complex forms of information. These are determined by the biological niche humans have constructed over evolutionary time. For example, understanding that a physical object retains its basic identity even when it changes shape or disappears would be a critical component of a cognitive system evolved to survive in a world in which there is are constantly moving objects. Similarly, and more in line with our current problem, understanding that objects that move by themselves can be considered to correspond to a category (which we refer to as implicitly living). Objects in such living categories are considered to have shared invisible attributes, which is a very useful way of conceptually dealing with the world in which separating out living from nonliving categories is a vital component, and understanding the specific properties of the latter can be particularly useful. Such a basically biologically based approach has in fact been proposed for human reasoning. As stated previously, Braine’s natural logic approach (Braine, 1978) takes just such a stance. There is one problem with this, however. The distinction between living and nonliving categories, and the ability to understand primitive transformational consistencies, such as those required to understand object permanence are found in very young children, which at least suggests empirical support for a biological hypothesis. This is simply not the case for inferential reasoning, and the idea that there is an essential biological basis that corresponds to some form of internal rules of inference appears to be empirically untenable.

Piaget’s approach to this problem was both biological and developmental (Piaget, 1971). In line with Kant, he assumed that the human mind did indeed require basic categories in order to adequately process information about an inordinately complex world. However, he did not assume that these categories were biological in origin. Instead, he postulated that biology provided the basic processes that allow systematic cognitive change. He also postulated that such changes were essentially systemic. In other words, he clearly made a distinction between the accumulation of knowledge, which could lead to a piecemeal and unconnected body of knowledge, and the development of the basic categories of mind that allowed people to process such knowledge. This latter can be considered the basis of the epistemology of the mind. In this perspective, the idea of a normative model of the mind can be seen as having the same basic function for cognition as the idea of a universal grammar has for language. More specifically, if human minds had essentially different epistemologies; that is, if they used different basic forms of categorization and reasoning, then the problem of just how people could communicate efficiently would arise.

Piaget added one component to this analysis. He started from empirical results that showed that children’s understanding of the world appears to progress through different levels or stages. He early on remarked that young children appear to have a more primitive epistemology than adults that is the underlying basis of their thinking relied on basic categories that were less consistent than those that appear to underlie adult reasoning. For example, young children have variable notions of the basic concept of quantity, being unable to consider that quantity is an invariant property of mass (Piaget et al., 1997). The lack of such invariance makes their thinking inherently unstable. A parent who is faced with a child who does not want to eat a meal because there is too much food, and who mushes up the food into a smaller area, is using this instability to successfully manipulate his or her child. In contrast, adults have no problem understanding invariance of quantity, in fact most consider the questions used to examine this notion to be simply stupid, since the answers are self-evident.

It is this form of basic difference in epistemology that Piaget attempted to describe in his research program, something that has often been lost in the debates over details about the age at which specific abilities appear, etc. This approach thus assumed that there was change and development in such a basic epistemology. However, the course of this development is not really variable, but was meant to mirror both the physical and biological properties that are critical components of the basic cognitive system. Thus, Piaget postulated that there was an invariant developmental sequence by which the very primitive cognitive categories present at birth, combined with whatever innate tendencies might drive early information processing, would gradually transform into the adult version. Piaget also supposed that epistemological development would tend towards the same basic normative model. A critical point is understanding just why this would be true. One reason, which underpins Piaget’s basic hypothesis is that epistemological structures are generated by interactions with the physical world. These start by interactions based on action and perception. Over time, and repeated interactions, children develop a logic of actions, which follows a coherent and nearly universal sequence (Piaget, 1965). In fact, this same sequence has been observed in primate species and some mammals (Scarr-Salapatek, 1976). Thus, it could already be argued that the end-point of sensori-motor development can be described by a normative model, one that reflects the deep structure of the way that humans structure physical action in the real world.

To make this distinction more explicit, when it is claimed that most 2 year-olds have the same basic epistemology, one that corresponds to a clear normative model, this does not mean that they have learned the same things. Specific learning clearly depends on the concrete environment in which children are raised. Thus a child might learn that cookies are good to eat in one context, and another might learn that candies are good to eat, depending on what is available. However, when trying to get cookies or candies or anything else that is desirable, that is hidden by an obstacle, all children will use the same action logic. Once again, the actual actions can vary (for example, one child might bat the obstacle away, while another might reach for it to move it away), but the logic of the action sequence is the same (action 1 is directed towards the obstacle with the aim of displacing it, action 2 is directed towards the goal). In fact, debates about sensori-motor cognition are mostly about whether sensori-motor logic is developed faster or slower, but there is little debate about the form of this logic. There are thus quite solid grounds for suggesting that development at this level can indeed be described by a normative model (Dasen, 1972).

Piaget’s explanation of development considers that more abstract forms of conceptually based cognition are derived by processes of representation and symbolic manipulation of the logic of actions. Thus, for example, basic categorization is derived from the process of perceptually based similarity relations, causal categories are derived from direct causality, etc. More specifically, reasoning reflects the basic structure of conditional action schemas. For example, there are many ways to get a biscuit, thus understanding the uncertainty of such actions directly reflects what children have already learned about the physical world (Byrnes and Overton, 1986). Increasingly abstract forms of reasoning require a long and complex process of representation and restructuring of more abstract concepts, but these still reflect the underlying structure of the physical world. This point of view would thus suggest that the end-point of epistemological development would be essentially the same. Once again, if one examines studies looking at the ability of pre-adolescents to make inferences about the concrete world, empirical results strongly suggest that the same level is attained by most children, although there is a great variation in age. Thus, most children can understand that liquid quantities are conserved over transformations, allowing them to consistently infer that a simple change in container will not alter the quantity.

The same is found with transitive inferences, the logic of which most children understand by adolescence when the content is concrete and clear (Markovits et al., 1995). Once again, similarly to studies of sensori-motor development, debate about the form of such concrete logic is not about the form of such logic, but about whether the corresponding abilities are developed more or less rapidly. Thus, there is also very clear evidence that the logic of pre-adolescents, which allows understanding of many forms of conservation, and basic forms of transitivity, causality, categorization, etc. when these are applied to concrete, perceptible problems consistently described the abilities (and performance) of most pre-adolescents (although age of acquisition is highly variable). In other words, a normative model can be claimed to exist to describe the concrete reasoning of most children by adolescence. Since development on the more abstract, formal level is derived from reasoning structures developed previously, this suggests that formal reasoning should indeed correspond to a single form of normative model. This is indeed the underlying rationale for Piaget’s claim that there is a single normative model for formal thinking.

## COMMUNICATION AND NORMATIVE REASONING

Having a shared epistemology is certainly useful in order to allow different members of a species to process variable information in ways that are internally consistent. There is one further argument for the existence of a single normative model of human reasoning. If this normative model is a model of the underlying epistemology of the conscious component of the cognitive system, then the ability to communicate must require sharing the same basic epistemology. In fact, it could be argued that normalization of System 2 reasoning is particularly critical in this context. In order to understand this point, it is useful to consider communication with System 1 (intuitive) reasoning. Although much of just what is involved in such reasoning remains mysterious, we can fruitfully speculate about some aspects of this. Intuitive inferences can reflect (at least) two forms of information. The most critical of these from our point of view is the internal structure of experiences stored in memory. Theories such as probabilistic models of inference (Oaksford and Chater, 2003) assume that intuitive inferences reflect stored knowledge about the world. A useful example is the belief-bias effect, which is the tendency of people to accept conclusions that are judged as believable as being logically valid (Evans et al., 1983). Although experiments examining this effect use conclusions that are highly believable for a large number of people, believability is clearly personal. In addition, there is certainly an emotional component to the force with which believability acts on System 2 processing. One excellent example of this is given by a study by (Klaczynski, 2000). He found that interference with System 2 processing was much stronger when the beliefs that were being examined were related to a domain with very high emotional valence (religion) than when these were related to a domain with lower emotional valence (class). There are increasing numbers of studies that link emotional experiences to inference-making (Blanchette and Richards, 2010). Thus, it is reasonable to assume that Intuitive processing tends to be highly personal, in that it reflects idiosyncratic personal experience, which conditions not only understanding of the underlying structure of experience and events, but is complicated by emotional valences that clearly reflect individual experiences. Intuitive processing is by definition unconscious, and certainly experience shows us that idiosyncratic and emotionally driven inferences do not generate much in the way of metacognitive awareness.

In other words, intuitive processing can clearly serve individual purposes by allowing people to make rapid, low-cost inferences that reflect their past experience. Doing so increases the chances that future behavior will mirror past circumstances, which allows individuals to profit from experience in a very immediate way. If that were the end of the story, there would be no need for any other inferential system. However, there is another component to human behavior, and that is the fact that humans live in social groups. This makes intercommunicability a critical component of any form of reasoning, since group behaviors require reconciling many divergent individual agendas in a way that allows group cohesion. Further complicating this dynamic is that fact that group complexity increases exponentially with numbers of group members. This explosion of social information has been hypothesized to be a major evolutionary driver for human cognition (Dunbar, 1993). Recently, Mercier (Mercier, 2011; Mercier and Sperber, 2011) has proposed a general evolutionary theory for the development of System 2 reasoning abilities that suggest that these abilities have evolved in order to regulate communication in complex social groups.

This perspective views reasoning as a form of argumentation which allows people both to present overt reasons for actions in an attempt to convince others and to allow others to evaluate arguments. While reasoning might have other functions, it is a useful hypothesis to see it as a means for exchanging explicit reasons for action, since this would have the potential to allow groups to make decisions that were more efficient than a simple majority rule would provide. Seeing reasoning as a form of communication, or even as a useful underpinning for communication, makes an even stronger case for the existence of a common epistemological core, since the essence of argumentation requires a sufficiently strong common basis.

Now, there are (at least) two ways that effective communication can be insured. The first involves the use of common biologically based intuitive schemas. In the absence of explicit language or symbolic thought, such schemas characterize the social cohesion of many social animals. There is reasonable evidence that humans also have such implicit social schemas. For example, we have recently shown that humans share an intuition about coalition formation as a function of individual power that is similar to what has been found behaviorally in chimpanzees (Benenson et al., 2009). Another form of implicit inference is that underlying the Gricean view of linguistic communication, in which pragmatic interpretations of language acts are underpinned by common assumptions that are derived from shared experience (Grice, 1981). However, such intuitive schemas can only work well when social behavior is relatively constrained. Human social behavior, while certainly sharing many aspects with more biologically constrained social species, is, however, very flexible. Flexibility, while allowing greater ability to adapt to changing circumstances, has a clear effect on the possibility of ensuring effective communication solely on the basis of shared intuitive schemas. This puts a greater functional burden on overt, language based communication to ensure that social interactions do not degenerate into conflicts based on differing individual intuitions and perceptions. But, in order for such communication to serve this function, it is imperative that there exist a shared epistemology, i.e., that the ideas shared overtly are underpinned by some common basic principles of reasoning. This again provides theoretical weight for the idea that explicit human reasoning should have a normative core.

## WHAT WOULD A NORMATIVE MODEL OF REASONING LOOK LIKE?

There are thus reasons related to the basic epistemology of human cognition and to the importance of reasoning to communication that suggest the necessity of a single normative model for explicit human reasoning. There are some basic considerations that can give clues to just what this model entails. The first is an argument from conceptual power. The last couple of hundred years have led to a proliferation of models of logic that have radically different underpinnings. Each of these models is a product of the human mind, individually or in concert with others. One important constraint for a normative model that does indeed correspond to the workings of the explicit, analytic mind is that it should allow the construction of multiple forms of models of logic. In addition, such a model should be readily understandable by most people, exactly because of the premise of communicability that we have claimed previously. Both of these constraints, along with historical and developmental considerations suggest that the normative model of the mind should involve some basic principles that underpin the notion of validity.

Secondly, one of the key components of cognitive development is that change goes towards increased complexity. A good example of this is well-documented in language learning, the phenomenon of over-generalization. Young children are faced with a complex variety of linguistic forms, with many idiosyncratic formulations which have historical roots, but often violate what are more frequent forms. Children’s strategies for learning language is to identify (by whatever process this is done) the most frequent pattern, and generalize this as a rule that is used in all occurrences of a given class, even when this involves generating words that have never actually been encountered (Onnis et al., 2002). Young children do the same with cognitive categories, picking out simple rules and extending these to concepts that are not actually instances of these categories. In other words, the developing human mind has a clear strategy, which requires generating simple rules and extending these to a wide variety of instances. It is only through continued interactions and reflection that these initial simple rules are extended to more complex concepts. What this in turn suggests is that if basic analytic reasoning relies on core normative principles, it will take a form that reduces the cognitive load required to reason. Thus, while there are a multitude of logical systems that take into account the true complexity of human experience, this argument suggests that normative principles will be less complex than any of these logics.

In this perspective, developmental studies can provide some very useful information. As we have seen, variability is a key characteristic of the reasoning of adults. However, while such variability is suggestive of a system of thought that has no common epistemology, the addition of an intuitive component of the functioning of the human mind makes variability more readily explicable in terms of the combination of a personal form of intuition which reflects individual experience and explicit, analytic thought that despite some variability has similar underpinnings.

The problem here is to determine exactly what are the critical components of normative thinking. The key to this is to distinguish between a normative model that is determined by its outcome, and one that is described by the nature of the underlying processes. Most studies that have looked at whether people reason normatively have examined outcomes, specifically whether the responses given to inferential problems are the same as whatever norm is being compared to. However, unless it is believed that inferential rules are constructed directly in the mind (as some theories do indeed suggest, e.g., Braine, 1978; Rips, 1983), we can rephrase the question of normality by trying to specify what kinds of underlying analyses must be implied by any system of thought that can in principle produce “correct” answers.

## CHILDREN’S UNDERSTANDING OF VALIDITY

In this perspective, we can distinguish at least two major components of basic logic that are necessary to produce a form of reasoning that can in principle become powerful enough to generate complex normative models. One important use of such a logic is the ability to explicitly examine the consequences of intuitive forms of reasoning in a way that allows people who do not share the same intuition to communicate. One key component of this ability is understanding the distinction between belief and some form of validity. In other words, before people can explicitly examine and compare the consequences of divergent personal experience, they must be able to distinguish, at least in principle, inferences that are derived directly from experience and those derived by some process of “logical reasoning”.

If this corresponds to a basic component of human reasoning, then it should be evident, in some form, in children. In fact, there is clear evidence that this distinction is fairly primitive. For example, Moshman and Franks (1986) found a clear developmental trend so that by early adolescence, most children can spontaneously understand the distinction between belief and validity, well before the level of schooling in which this distinction is taught. More strikingly, Morris (2000) found that children as young as 5-years of age, when given an appropriate content can generate this distinction. In other words, understanding the distinction between belief and validity is an early developmental acquisition, a critical one if explicit reasoning is indeed a counterpoint to intuitive reasoning. Of course, this is not really news to parents of young children, who despite the real difficulties of doing so, are nonetheless able to “reason” with children in a way that confronts the child’s intuitions with some form of logic.

## POSSIBILITY AND NECESSITY: COUNTEREXAMPLES IN REASONING

If children can understand the distinction between validity and belief, the next question is just how validity is determined. This of course goes to the question of just what kind of “logic” is available to children, and how this is related to the logic of adults. I have claimed (Markovits, 1993) that the key component of understanding this question derives from one of Piaget’s later works, on the relation between possibility and necessity (Piaget, 1987).

Before examining this, it is useful to make an important distinction underlying Piaget’s approach. Piaget proposed standard propositional logic as a competence model for advanced adult reasoning. This has often been interpreted as implying a rule-based form of reasoning, which would invariably lead to standard logical responses. However, this is a mischaracterization. What was specifically proposed was that the underlying epistemology that characterized advanced adult reasoning would allow the ability to generate such responses. The work on possibility and necessity was an attempt to specify the nature of this epistemology. The basic question that was raised concerns the kinds of factors that can explain how children and adults can conclude that a potential conclusion is necessary. The analysis makes no mention of rules, instead it places such logical conclusions within the more general context of the range of information that can be generated by the reasoner. In this, one critical component of reasoning is the range of possibilities that are processed by children in the context of a given problem. A given inference is necessary if it excludes whatever possibilities are generated by the child at the moment of reasoning. This corresponds to what one could refer to as local necessity, since the actual generality of a conclusion depends critically on the range of possibilities that are generated. Critically, if a child or adult is aware of a possibility that is not excluded, they will reject a given conclusion. This interaction between possibilities and necessity is modulated by the degree of abstraction of the processes used to analyze a given problem.

A similar idea underlies the mental model analysis of reasoning (Johnson-Laird, 2001; Johnson-Laird and Byrne, 2002). Mental model theory considers that people construct internal representations of possible states of the world (models) that characterize the major premise of a given inference. Possible states must be generated by a reasoner, based on combinations of semantic and pragmatic factors (Byrne, 2005). Critically, an inference is considered to be valid if there are no explicit counterexamples in the reasoner’s representation. The presence of a counterexample is sufficient to render a putative conclusion invalid. An inferential judgment is thus an on-line consequence of a reasoner’s ability to (1) generate a more or less full range of possibilities consistent with premises and (2) determine whether these possibilities contain a counterexample.

One important distinction between the Piagetian analysis and mental model theory is that the latter postulates that internal representations are derived from a semantic analysis of logical connectors, albeit one that is modulated by pragmatics. Since the semantics of logical connectors are assumed to be generally invariant, variation in reasoning performance is accounted for by such individual difference factors as memory capacity. Mental model theory does not have a very clear developmental component, although Barrouillet and colleagues (Barrouillet and Lecas, 1999; Barrouillet et al., 2008) have suggested that working memory limitations can affect children’s ability to actually represent the full semantics of logical connectors. Development will necessarily tend towards the same forms of logical reasoning that are determined by the shared semantics of logical connectors. Thus, although mental model theory presents an analysis of reasoning that is consistent with the interaction between possibility and necessity, the developmental component has a very different focus (see Markovits and Barrouillet, 2002 for a version of mental model theory that has a developmental focus that is more consistent with the Piagetian model).

Piaget’s work indicated that one of the key factors in development is the ability to generate increasingly abstract forms of possibilities (Gauffroy and Barrouillet, 2011). Young children start by considering possibilities that are more concrete and related to the situational factors for a given problem context. With development, these possibilities become more extended and abstract, and less tied to situational constraints. However, by 6- or 7-years of age (Markovits, 2000), children can understand that a conclusion that eliminates all other possibilities is necessary. Of course, since this judgment of necessity depends on the range of possibilities that are eliminated when this conclusion is made, it is subject to revision even if exactly the same form of reasoning is applied, since the generation of possibilities can vary from one moment to the next. In other words, reasoning at this level is sequentially defeasible (Pollock, 1987), that is the same person can arrive at a different conclusion for the same inference, simply because the domain of possibilities accessed during reasoning might change, see Markovits (1985) and Byrne (1989) for examples of defeasible reasoning in adults. However, the *processes* by which judgments of validity are made are in principle general. More importantly, it is possible to overtly challenge any such inference by comparing possibilities. This in turn is realistic only because judgments of validity depend, not on an accumulation of data, but on the presence or absence of a counter-example to a given conclusion.

There is one further point that can be made in this context. As noted previously, it has been argued that most relations are, in real life probabilistic (Oaksford and Chater, 2007). If the point of reasoning was to faithfully reflect the real characteristics of the real world, then one would expect reasoning to be essentially probabilistic. However, the analysis of “logical” reasoning that I use here does not generate conclusions such as “it is probably true that….” It simply allows judgments of validity or not, in other words, it allows concluding that something is certain or that it is not. Why should this form of judgment be useful when trying to reasoning about phenomena that are inherently probabilistic? There are some good reasons for this, but the chief one is that it is cognitively much simpler. While a probabilistic judgment or any other kind of intuitive judgment that relies on stored knowledge about the world can conceivably be made very rapidly by associative processes, explicitly communicating the basis for such judgments would in theory require explicitly processing a large quantity of information. In fact, in many cases most people are unable to do such explicit processing. In this, case comparing conclusions would simply result in people fighting over personal beliefs. Even if we assume that a reasoner does (remarkably) have conscious access to the relevant information, and is consciously aware that conclusion X is probable because of data set Y, the problem of how someone else, whose personal data base does not contain the same information, can process this conclusion.

A useful, because somewhat more real example, can be taken from research on aggressive behavior. One of the underlying mechanisms of such behavior concerns the expectation (mental model) that a given social interaction will have an aggressive outcome (Dodge et al., 1990). Children who develop a strong expectation that interactions will be aggressive, tend to infer that most actions will have an aggressive outcome. Now, imagine that two children with differing expectations are interacting, and attempting to determine the outcome of a given course of action. In order to make a reasonable comparison, both children would have to recount the many kinds of interactions that form the basis of their expectations. Then each would have to attempt to integrate the other’s information into their own internal data base, a daunting task at best, and one that would require not only a great deal more cognitive resources that most children possess, but a great deal of time. The fact that such discussions do not really take place might well account for the difficulty in lowering aggressive behavior (Dishion et al., 1999). In contrast, since many arguments consist in deciding on taking a given course of action or not, a simple counterexample strategy would be quick and useful, precisely because of its inherent limitations. If this is indeed characteristic of basic reasoning, then once again, developmental studies should provide evidence.

## THE DEVELOPMENT OF ELEMENTARY CONDITIONAL REASONING

We can illustrate this evidence by examining simple conditional (if–then) reasoning. Conditional reasoning is one of the key components of propositional logic, and is certainly one, if not the most, frequently studies forms of reasoning. Piaget considered that conditional reasoning was one of the key competencies of formal operational thinking. The basic approach that we are taking here suggests that understanding the development of conditional reasoning will rest upon the basic understanding of the interplay between possibility and necessity and the way that this can be used increasingly abstract context. The key component of this suggestion is identification of just what the range of possibilities are required in order to make “logical” conditional inferences, and whether children are able to process these in a way that allows them to make simple judgments of validity.

To simplify this discussion, we focus on two conditional inferences. The Modus ponens (MP) inference involves reasoning that “If P then Q, P is true.” The affirmation of the consequent (AC) inference is “If P then Q. Q is true.” The logical conclusion to a MP inference is that “Q is true”. Now studies with adults have shown that the ability to conclude that the MP inference is valid depends on the extent to which disabling conditions (Cummins et al., 1991; Cummins, 1995) are incorporated into people’s representations of premises. A disabling condition (disabler) is a condition that is associated with a given P then Q premise, one that could invalid the link between antecedent and consequent terms. The strongest form of disabler is given when reasoning with empirically false premises, for which the true relation is a disabler. Very young children have little problem in considering MP inferences to be valid for a variety of contents. The one major exception is reasoning with empirically false premises, for which young children have great difficulties in accepting the MP inference. However, interventions that allow them to inhibit retrieval of the true disabler, such as embedding premises into a fantasy context (Dias and Harris, 1988, 1990) dramatically improve their ability to accept this inference. With increasing age, children are more able to spontaneously inhibit potential disablers when given standard logical instructions (Markovits and Vachon, 1989).

In contrast, there is no logical conclusion to an AC inference, although typically, many people respond to AC inferences by concluding that “P is true” (Cummins et al., 1991; Thompson, 1994). The reason for the theoretical lack of any logical conclusion is that a true conditional allows for the possibility of what have been referred to as alternative antecedents, that is cases where alternate conditionals “If A then Q” are, or might be true. Considering such alternatives (which comprise the domain of possibilities in this case), allows producing a counterexample to what is the usual AC conclusion. In fact, there is strong empirical evidence that the availability of such alternative antecedents in memory is a strong determinant of the conclusions given to AC inferences (Markovits and Vachon, 1990; Cummins et al., 1991; Thompson, 1994; Quinn and Markovits, 1998). In other words, when people can access such alternatives when reasoning, they tend to reject the certain conclusion for AC inferences.

Analysis of both the MP and the AC inference shows that, in both cases, children’s judgments of validity are determined by the kind of information that is incorporated into the representation of premises, during the online process of reasoning. Although the specific information varies (disablers and/or alternative antecedents), the basic dynamic is the same. Even young children will accept a conclusion as valid if their representation of the premises does not include a potential counterexample, otherwise the conclusion will be considered to be invalid. Finally, although considering these two inferences is particularly useful, it is worth briefly examining the two other inferences that define conditional logic. Studies have generally shown that responses to the denial of the antecedent (DA) inference responds to the presence or the absence of alternative antecedents in the same way as AC inferences. The developmental pattern is also very similar. Similarly, the modus tollens (MT) inference corresponds somewhat similarly to the MP inference to the presence of disablers. However, the use of negation in both these inferences somewhat complicates analysis. Understanding the basic notions underlying the understanding of logical validity is more easily presented by concentrating on the MP and the AC inferences.

I have in fact argued that the best elementary definition of “logical” reasoning is the ability to understand the certainty of the MP inference and to simultaneously understand the uncertainty of the AC inference (Markovits and Lortie Forgues, 2011). As I have stated, the key factor in this form of reasoning is the kind of information that is incorporated into children’s representations of premises. Constructing a representation of premises that does not include potential disablers, but that does include potential alternative antecedents requires inhibiting the former while retrieving the latter. The tension between these two contradictory cognitive processes can explain why even educated adults find it difficult to reason logically in the limited sense that we use (see Markovits, 2014). However, there is strong empirical evidence that children as young as 6–7-years of age are indeed able to understand both the certainty of the MP inference and the uncertainty of the AC inference, when the content of conditional premises allow for very ready access to alternatives. For example, when reasoning with propositions such as “If an animal is a dog, then it has four legs. An animal has four legs. Is it a dog?,” young children will readily reject this inference (Markovits, 2000). More tellingly, they will do so by explicitly citing the existence of a counterexample to the implied conclusion (“cats have four legs”). When given problems where the alternatives are explicitly presented, even younger children will reject the AC inference while accepting the MP inference (Markovits and Thompson, 2008).

In other words, very young children are able to reason “logically” with simple, direct forms of propositional logic when the content allows appropriate inhibition and retrieval of relevant information. Given the strong tendency of children to accept inferences, the most striking part of these results is that they are capable of processing the fact that there are alternatives to a putative conclusion, and using this as a basis for rejecting a putative conclusion. I would then argue that this is exactly the nature of the kind of logical reasoning that is the “norm” for most people, which involves constructing a simple representation of an inference and deciding on the validity of a potential conclusion based on the existence of a possible counterexample.

Does this model imply that children or even educated adults will consistently give the standard propositional logic answer to (for example) all AC inferences. Not at all. Since the exact response to any inference depends on the range of counterexamples that are generated while reasoning, which is in turn related to retrieval and inhibitory processes, variability is expected. This is an important point to make. The increasing ability to generate potential counterexamples can in fact produce variable responses. For example, children who are more efficient in simply producing potential causal alternatives tend to reject the MP inference more frequently (Janveau-Brennan and Markovits, 1999). This corresponds to the notion of local necessity, in which validity is the result of application of the basic principle related to counterexample detection to an online process of possibility generation. Thus, this model makes no presuppositions about the nature of the underlying rules or algorithms. Such a basic epistemology is thus potentially consistent with a wide variety of reasoning systems, including for example the incomplete logic proposed by Gauffroy and Barrouillet (2009).

The important part of this conception of reasoning is the idea that people can recognize the presence or the absence of a counterexample and use this to make a judgment of validity that is internally consistent. Critically, especially for its usefulness in communication, people can adjust their inferences based on externally communicated counterexamples. Evidence shows that both children (Rumain et al., 1983) and adults (Markovits, 1985) will revise conclusions to AC inferences when given additional information about potential alternative antecedents. Similarly, explicit information suggesting disablers results in adults rejecting the MP inference (Byrne, 1989). The effect of providing explicit information about the existence of potential counterexamples is to reliably increase rates of rejection of conclusions that were previously accepted. In other words, both children and adults are able to revise conclusions that are generated by their internal inferential processes simply by considering alternatives furnished by other people. In a similar vein, Klaczynski (2001) has shown that children are able to recognize logical arguments that rely on the presence of counterexamples as being superior to arguments that rely on other kinds of processes.

In this context, basic logical reasoning can be seen as the ability to use a counterexample to invalidate an otherwise acceptable conclusion. Empirical results show that this ability is present at a very early age. Critically, there is also evidence that both children and adults can use externally presented counterexamples to modify their own inferences. Thus logical reasoning has both an internal function that allows for judgments of validity and an external function that provides a basis for modifying personal judgments by considering specific arguments that present potential counterexamples.

If such an ability does indeed represent the kernel of logical reasoning, then development should reflect not any change in this basic form of logic, but should be tied to the increasing ability to generate and inhibit potential counterexamples in an increasingly abstract way. I have indeed argued that this is a good description of the development of simple conditional reasoning between early childhood and later adolescence (Markovits, 2013). To summarize this pattern, young children can consistently reject the AC premise and accept the MP premise for premises that use if–then relations that link classes and properties (Markovits, 2000). The ability to do the same with causal conditionals (“If cause P then effect Q”) is found only in pre-adolescents (Janveau-Brennan and Markovits, 1999). Reasoning this way with contrary-to-fact premises is a later development (Markovits and Vachon, 1989). Finally, understanding the certainty of the MP inference and the uncertainty of the AC inference with completely abstract premises is a much later development, and is only found with educated adults (Venet and Markovits, 2001; Markovits and Lortie Forgues, 2011). In other words, the developmental pattern is completely consistent with the idea that the basic mechanism underlying simple logical reasoning is available to quite young children, and that subsequent development extends this same process to increasingly complex and abstract forms of content.

Importantly, extending basic reasoning to abstract content (Markovits and Lortie Forgues, 2011) gives the ability to reason logically even with content that has no concrete referents or for which the referents are unintuitive. Such a form of reasoning is the historical basis for the various models of logic that have been constructed, and which correspond to a wide variety of different forms of reasoning. In other words, the kind of abstract reasoning that develops from the primitive base found in young children can become powerful enough to allow people to construct normative models of many types.

## Conflict of Interest Statement

The author declares that the research was conducted in the absence of any commercial or financial relationships that could be construed as a potential conflict of interest.
